# The amygdala lesioning due to *status epilepticus* – Changes in mechanisms controlling chloride homeostasis

**DOI:** 10.1016/j.clinsp.2022.100159

**Published:** 2023-02-10

**Authors:** Luiz E.C. Santos, Antônio-Carlos G. Almeida, Sílvia C.B. Silva, Antônio M. Rodrigues, Samyra G. Cecílio, Carla A. Scorza, Josef Finsterer, Marcelo Moret, Fulvio A. Scorza

**Affiliations:** a Laboratório de Neurociência Experimental e Computacional, Departamento de Engenharia de Biossistemas, Universidade Federal de São João del-Rei (UFSJ), São João del-Rei, MG, Brazil; b Disciplina de Neurologia Experimental, Escola Paulista de Medicina (Unifesp), São Paulo, SP, Brazil; c Krankenanstalt Rudolfstiftung, Vienna, Austria; d SENAI ‒ Departamento Regional da Bahia, Centro Integrado de Manufatura e Tecnologia, Bahia, BA, Brazil

**Keywords:** Amygdala, SUDEP, Non-synaptic mechanisms

## Abstract

**Objective:**

Amygdala has been demonstrated as one of the brain sites involved in the control of cardiorespiratory functioning. The structural and physiological alterations induced by epileptic activity are also present in the amygdala and reflect functional changes that may be directly associated with a sudden unexpected death. Seizures are always associated with neuronal damage and changes in the expression of cation-chloride cotransporters and Na/K pumps. In this study, the authors aimed to investigate if these changes are present in the amygdala after induction of *status epilepticus* with pilocarpine, which may be directly correlated with Sudden Unexpected Death in Epilepsy (SUDEP).

**Methods:**

Pilocarpine-treated wistar rats 60 days after *Status Epilepticus* (SE) were compared with control rats. Amygdala nuclei of brain slices immunostained for NKCC1, KCC2 and α_1_-Na^+^/K^+^-ATPase, were quantified by optical densitometry.

**Results:**

The amygdaloid complex of the animals submitted to SE had no significant difference in the NKCC1 immunoreactivity, but KCC2 immunoreactivity reduced drastically in the peri-somatic sites and in the dendritic-like processes. The α_1_-Na^+^/K^+^-ATPase peri-somatic immunoreactivity was intense in the rats submitted to pilocarpine SE when compared with control rats. The pilocarpine SE also promoted intense GFAP staining, specifically in the basolateral and baso-medial nuclei with astrogliosis and cellular debris deposition.

**Interpretation:**

The findings revealed that SE induces lesion changes in the expression of KCC2 and α1-Na+/K+-ATPase meaning intense change in the chloride regulation in the amygdaloid complex. These changes may contribute to cardiorespiratory dysfunction leading to SUDEP.

## Highlights

• Amygdala is involved in the control of cardiorespiratory functioning.
• KCC2, α_1_-Na^+^/K^+^-ATPase and GFAP changes were observed in the amygdala after *SE*.
• The changes in chloride regulation may contribute to SUDEP.

## Introduction

Recently, a functional connection between the amygdala and respiratory control in people with TLE was demonstrated, providing evidence that conscious mouth breathing prior to stimulation may prevent apnea.[1] Importantly, it has been demonstrated that specific amygdaloid nuclei may be at the center of this process. The study also shows that the attentional state is critical to apnea mediated by amygdala activation.^1^,^2^

The occurrence of epileptic seizures is always associated with neuronal damage and changes in the expression of the cation-chloride cotransporters,^3^,^4^ and also of the Na/K pump,^5^,^6^ to whom the cotransporters' functioning is intrinsically connected. These mechanisms play a crucial role in the ionic balancing between the intra and extracellular spaces,^5^ which, in turn, may modify the proper activity of the brain regions affected.

Based on this information, this study aims to verify if pilocarpine-induced status epilepticus causes neuronal damage and changes in the expression of NKCC1, KCC2 and Na/K-ATPase in the amygdala. As the anatomical substrate of epileptic activity in the central nervous system shows a direct relation to cardiovascular alterations, it has been proposed that patients with drug-resistant epilepsy associated with Lateral-Posterior thalamic nuclei (LP) lesions may face a particular risk of SUDEP.^7^ Moreover, studies showed that Temporal Lobe Epilepsy (TLE) can be related to atrophic changes in brainstem regions involved in central autonomic control that could be responsible for interictal and ictal autonomic abnormalities which can aggravate the damage to critical parts of the autonomic control system and thus potentially increase the risk for SUDEP.^8^,^9^ However, the exact mechanisms linking epilepsy with SUDEP still remain unknown and the present work aims to contribute to this question by examining related changes in mechanisms responsible for ionic homeostasis, which is directly involved with neuronal activity.

## Methods

### Ethical approval

All experiments were performed according to the guidelines established by the Ethical Committee for Experimental Use of Animals of the Federal University of São João del-Rei (Protocol nº 002/2015). The rats were housed under controlled conditions (ambient temperature 22‒24°C, 50% relative humidity, and lights on from 7 A.M. to 7 P.M.) with water and food available *ad libitum*.

### Induction of epilepsy

Male Wistar rats (230±30g; 50 days old) were randomly divided into 2 groups: control (C, n=6) and epileptic (E, n=20). Group E was submitted to a protocol for inducing experimental temporal lobe epilepsy by i.p. injection of a cholinergic agonist pilocarpine hydrochloride (360 mg/Kg, Sigma), after 20 min of pre-treatment with scopolamine methyl nitrate (1 mg/Kg, Sigma) by subcutaneous injection, in order to restrict peripheral cholinergic effects.^10^ Systemic administration of pilocarpine induces electrographic and behavioral seizures that progress to Status Epilepticus (SE) and may last several hours. Diazepam (10 mg/kg, Teuto) was administered 2 hours after SE in order to limit the several behavioral effects. From SE, a latent period characterized by progressive normalization of behavioral and electrographic effects lasts up to 7 weeks, followed by a chronic period marked by the appearance of spontaneous recurrent seizures that resemble human partial complex seizures.^11^ The animals from group E that survived the protocol (n = 6) were maintained for 60 days, after SE induction, under continuous monitoring from a motion detection system and nocturnal infrared light. The observed mortality in the treated group is in accordance with an age-dependent mortality determined for the pilocarpine model.^12^ All animals used in this study had at least two episodes of recurrent spontaneous seizures with a minimum interval of 24 hours between seizures, observed on the last day of monitoring. After this period, the animals were assigned to autoradiography and immunohistochemistry.

### Immunohistochemistry staining

The animals of groups C (n = 6) and E (n = 6) were euthanized with anesthetic overdose (ketamine-xylazine, 100 mg/kg ‒ 5 mg/kg, respectively) followed by transcardial perfusion with phosphate-buffered saline (0.1 M diluted in PBS; pH=7.4) and paraformaldehyde 2% in PBS 0.1 M. The brains were removed and immersed overnight in paraformaldehyde 2%. After post-fixation, brains were bathed in PBS and incubated at 4°C for posterior sectioning (40 µm thick slices) using a vibratome (Leica Microsystems, Germany).

Coronal slices of the amygdaloid nucleus, from rostral to caudal (-2.8–3.8 mm), taking the bregma as a reference,^13^ were bathed for 4 hours with blockage solution for unspecific sites (10% albumin serum bovine in PBS 0.1 M and 0.3% Triton X-100), at a room temperature. Following, slices were incubated for 48h, at 4°C, in solution (2% albumin serum bovine in PBS 0.1 M plus 0.1% Triton X-100) containing anti-KCC2 primary antibody (rabbit polyclonal antibody; 1:500; Abcam) e anti-α_1_-Na^+^/K^+^-ATPase (a6f-c, mouse monoclonal antibody; 1:100; Departmental Studies Hybridoma Bank) or anti-GFAP (rabbit polyclonal antibody; 1:1000; Abcam) and anti-NKCC1 (mouse monoclonal antibody (T4, 1:100; Departmental Studies Hybridoma Bank). After, sections were incubated for 2h, at room temperature, in a solution containing secondary antibodies anti-IgG rabbit (goat polyclonal conjugated with DyLight® 488; 1:250, Abcam) and anti-IgG mouse (goat polyclonal conjugated with DyLight® 594, 1:250; Abcam). In order to test for antibody specificity and possible tissue autofluorescence interferences, some slices were subjected to incubations where the primary antibodies, or both, were omitted. Sections were mounted on glass slides and covered with coverslips deposited using glycerol.

The quantitative analysis of the immunoreactivity intensity of NKCC1, KCC2, GFAP and α_1_-Na^+^/K^+^-ATPase were performed considering the optical absorption. The photomicrographs (n=6, per amygdaloid nucleus, for each hemisphere) were captured with a confocal microscope (Zeiss LSM 710) equipped with primary beam splitter 488/543, with argon laser 488 nm for the Dylight® 488 conjugated secondary antibody fluorophore and helium-neon laser 543 nm for the Dylight 594® conjugated secondary antibody fluorophore. The images were captured with a 63× glicerol immersion objective (Plan-Apochromat 63×/1.40 Oil Dic M27). The pinhole was adjusted to 1 airy unit.

### Optical densitometry

The optical densitometry analysis was performed to quantify the immunoreactivity intensity for NKCC1, KCC2, GFAP, and α_1_-Na^+^/K^+^-ATPase. Three brain slices per animal containing the amygdaloid nuclei were sampled from both hemispheres. From each staining were captured 6 photomicrographs of the amygdaloid nuclei using the 63× objective. The confocal photomicrographs were processed in RGB and compressed for the grayscale (RGB mean band) in order to obtain the corresponding histograms. To enhance the contrast, an equalization method was incorporated into the analysis.^14^ Moreover, to avoid autofluorescence interference due to the lipofuscin depositing in neurons these regions were digitally subtracted from the collected photomicrographs. The resulting images allowed enhancing the staining and, therefore, resulted in more reliable segmentation. A grayscale interval (from 0 to 65) was defined as the threshold for delimiting the immunoreactive area of the tissue. The significant pixels were posteriorly converted in a binary matrix (black and white) and quantified by the summation of the black pixels of the area. The quantification was performed with a computational system developed in MATLAB. The data were plotted as a percentage of the equivalent immunoreactivity of each photomicrograph.

### Statistical analysis

The mean intensity of the immunoreactivity for NKCC1, KCC2, GFAP, and α_1_-Na^+^/K^+^-ATPase from the amygdaloid nuclei of the control and epileptic groups was analyzed. The Shapiro-Wilk test was applied for inspecting the sampling distribution. Since all data analyzed followed a normal distribution, the Student's *t*-test was used for unpaired samples (unp-TT). All data were shown in mean ± Standard Error of the Mean (SEM). In all cases was adopted the significant level of 5%.

## Results

### NKCC1, KCC2 and α_1_-Na^+^/K^+^-ATPase immunoreactivity changes in the amygdaloid nucleus 60 days after SE

**Fig. 1:**
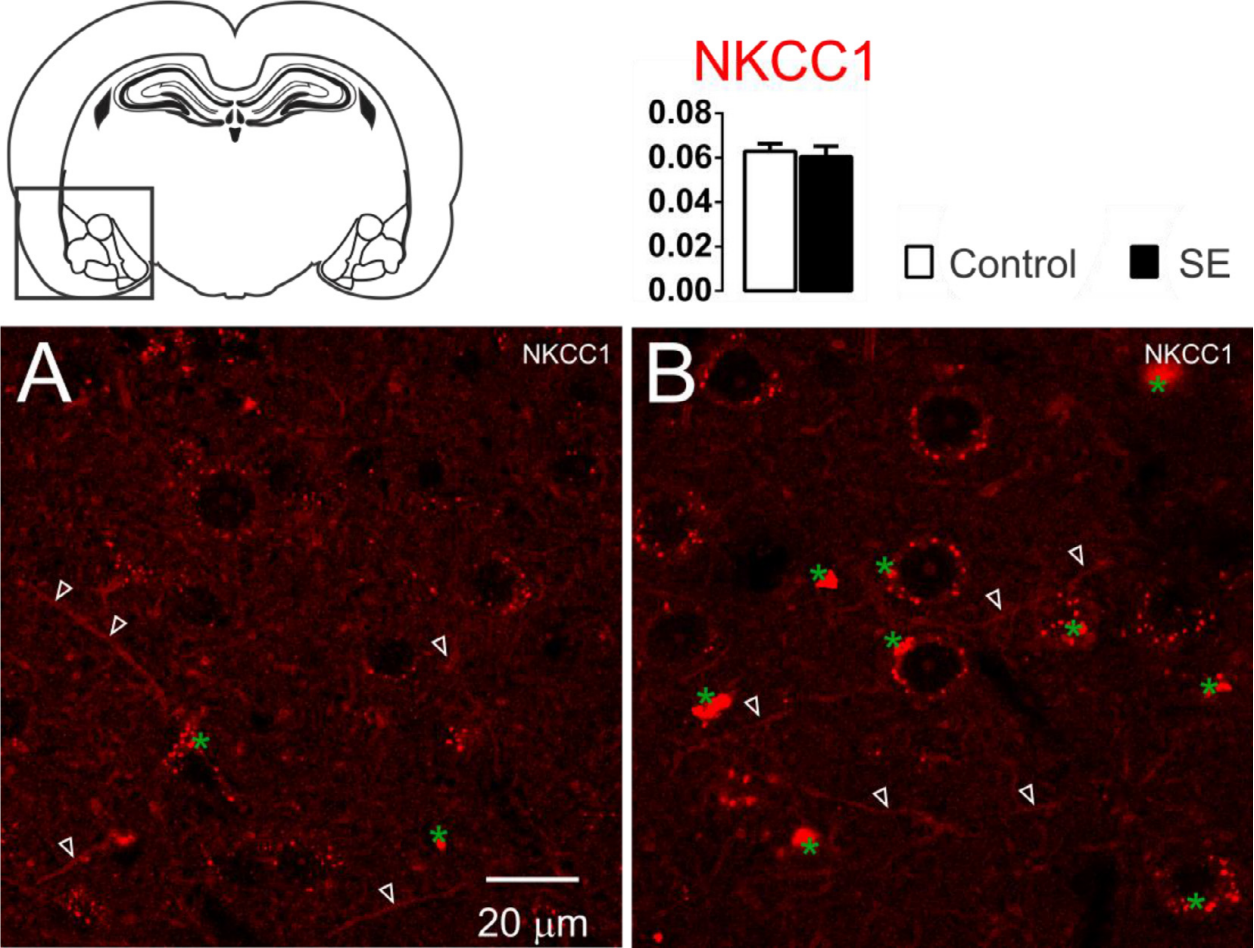
Representative photomicrographs of the NKCC1 immunoreactivity. The NKCC1 staining is limited to the dendrites processes (white open arrowheads). No significant difference was observed between groups C (1A) and E (1B). Data are means ± SEM. Green asterisks show agglomerates of lipofuscin. Note that lipofuscin granules are larger and more compact in the amygdaloid nuclei shown in 1B than those shown in 1A.

The NKCC1 immunoreactivity in the amygdaloid nuclei was limited to the dendritic-like processes. Few or almost absent staining was observed in the peri-somatic or perinuclear regions (Fig. 1). No significant differences were observed in the analysis of optical densitometry of the NKCC1 immunoreactivity of the tissues associated with groups C and E (C: 0.063 ± 0.003; E: 0.061 ± 0.004; *t* = 0.428; df = 10; p = 0.6776, unp-TT).

**Fig. 2:**
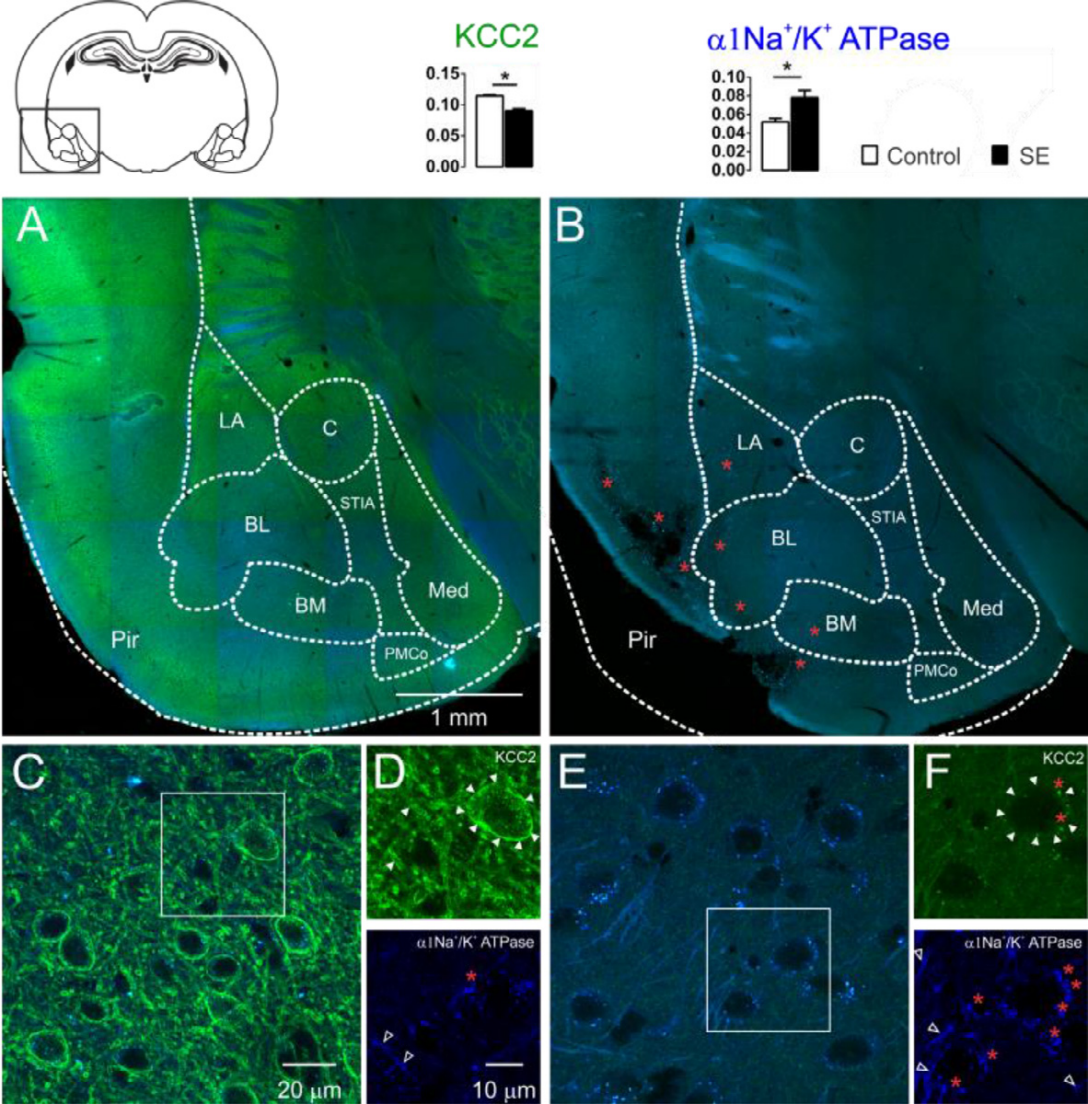
Confocal images of the immunostaining for KCC2 (green) and α1-Na^+^/K^+^-ATPase (blue) of the amygdaloid nuclei of the groups C (2A, 2C e 2D) and E (2B, 2E e 2F) and the corresponding percentages of pixels stained according to optical densitometry for both groups (LA, Lateral Nucleus; BL, Basolateral Nucleus; BM, Basomedial Nucleus; PMCo, Posteromedial Cortex Nucleus; C, Central Nucleus; Med, Medial nucleus; Pir, Piriform cortex nucleus; STIA, *Stria Terminalis*). Even in the images of lower magnification, it is possible to observe the intense reduction of the KCC2 immunoreactivity of group E (2B) compared with group C (2A). This is clear for higher magnification: group C (2C and 2D above, closed arrowheads) with intense peri-somatic staining for KCC2, which is also intense in the dendritic processes of the amygdaloid compared with the photomicrographs of the group E (2E and 2F above, closed arrowheads) where the staining is reduced. However, the α1-Na^+^/K^+^-ATPase immunoreactivity was intense in the amygdaloid nucleus of the group E (2E and 2F below, open white arrowheads) when compared with the images of group C (2C and 2D below, open white arrowheads). The red asterisks indicate regions with intense lipofuscin agglomerate, low (2B) and high magnification (2D e 2F). Note large, lesioned region of the paralimbic cortex (2B), more specifically the piriform cortex. Optical densitometric data given in mean ± SEM, (*p < 0.05).

The KCC2 immunoreactivity was intense in the control group. In the entire extension of the investigated amygdaloid nuclei, as well as in the dendritic-like processes, intense peri-somatic staining was observed. In contrast, slices of group E showed a drastic reduction in the staining intensity. Neurons of the amygdaloid nuclei of the epileptic animals had weak contour delimitation of the somatic plasmatic membrane; therefore, the typical peri-somatic staining was not present. Moreover, a few dendritic-like processes could be seen with intensity comparable to the control group. Typical images of the KCC2 immunoreactivity staining of the amygdaloid nuclei comparing control and epileptic groups are shown in Fig. 2. From the quantitative analysis of the KCC2 staining, the optical densitometry exhibited a significant reduction of the KCC2 expression in the group E (C: 0.115 ± 0.002; E: 0.090 ± 0.003; t = 6.693; df = 8; p = 0.0002, unp-TT).

It is known that the ionic transport mediated by the cotransporters expends energy derived from the transmembrane ionic gradient generated by the Na^+^/K^+^-ATPase, therefore, indirectly, the energy is derived from the ATP hydrolysis.^3^,^5^ Since changes in the Na^+^/K^+^-ATPase expression will also affect the cotransporters' action, it is necessary to investigate changes in the expression of this enzyme to interpret the changes in the expression of the cotransporters. For this aim, the authors investigated the changes in the immunoreactivity of the α_1_-Na^+^/K^+^-ATPase subunit in the amygdaloid nuclei of the slices of each group studied. Qualitatively, the α_1_-Na^+^/K^+^-ATPase subunits revealed the dendritic-like processes in three different types of staining: evident, weak, and absent peri-somatic staining. As shown in Fig. 2, the staining of group E was more intense than the staining of group C. This observation was confirmed statistically by comparing the optical densitometry (C: 0.052±0.003; E: 0.078±0.007; t=3.112; df=8; p=0.0144, unp-TT).

The specificities of the cotransporters and α_1_-Na^+^/K^+^-ATPase immunoreactivity were evaluated from the negative controls omitting the primary and/or the secondary in each group of slices. In this case, staining similar to that observed in the two groups was not found. However, auto-fluorescent intra and extracellular granules characterized by small globular aggregates were revealed in an extensive band of excitation with far red emission. These aggregates are similar to lipofuscin, a common pigment observed in mature neurons, formed from organelle debris, remains from cellular metabolism or oxidative stress, which accumulates in the cytoplasm as time evolves.^15^ Small points similar to lipofuscin, distributed around the nuclei, in the cytoplasm, were observed in control slices. However, in the slices of group E, the aggregates observed were larger and occupied plenty of the area in the cytoplasm. In addition, cells with smaller nuclei (supposedly glia) had voluminous auto-fluorescent aggregates near the nucleus (Figs. 1 and 2).

### Astrocytic immunoreactivity of the amygdaloid nuclei 60 days after SE

**Fig. 3:**
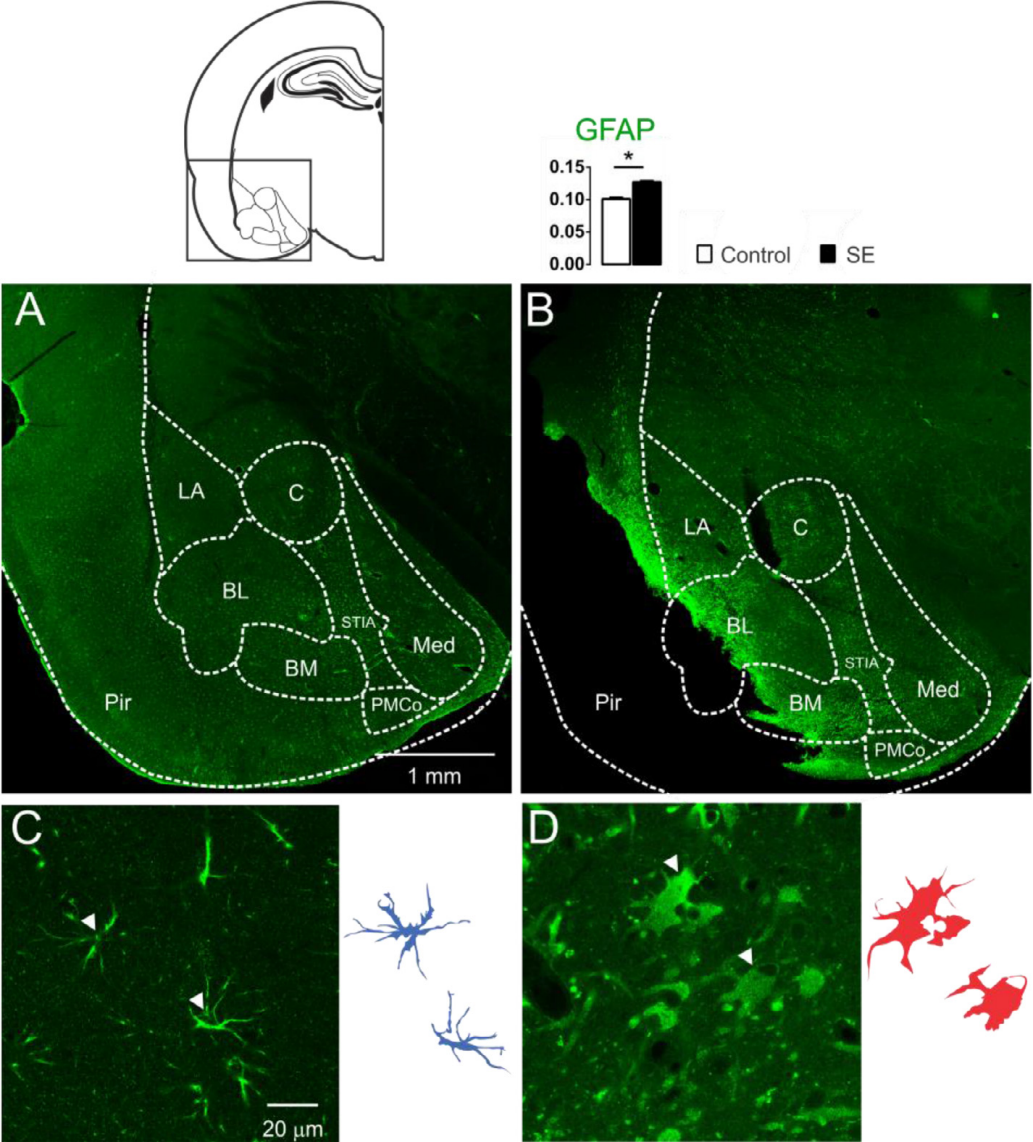
Confocal images of the immunoreactivity for GFAP of the amygdaloid nucleus, groups C (3A and 3C) and E (3B and 3D). In 3A, normal astrocytic morphology in the amygdaloid nucleus was observed in all control animals. However, as shown in 3B, all slices of group E showed intense astrocytic agglomerates with strong immunoreactivity near the lesioned regions, like Pir, BL and BM (LA, Lateral Nucleus; BL, Basolateral Nucleus; BM, Basomedial Nucleus; PMCo, Posteromedial Cortex Nucleus; C, Central Nucleus; Med, Medial Nucleus; Pir, Piriform Cortex; STIA, *Stria Terminalis*). Photomicrographs in high magnification show the astrocytes' morphological differences when comparing group C (3C) with group E (3D). In 3D, the astrocytes appear corpulent and with thick and short segments, which are typical of reactive astrogliosis. Astrocytes reconstruction for the control is shown (3C-right) and can be compared with the reconstruction for group E (3D-right). Normal astrocytes (in blue) have thin segments and protoplasmatic morphology. The optical densitometry confirms the GFAP immunoreactivity increase compared with the group C. Data given in mean ± SEM, (*p < 0.05).

The presence of cells with large aggregates of lipofuscin, after 60d of SE, generated the hypothesis of glial reactivity, which can occur in regions of the nervous system with intense tissue injury. With these purposes, the presence of the glial reactivity was evaluated in the amygdaloid nuclei by means of investigating the GFAP immunoreactivity of the animals 60 days after SE. Intense GFAP staining was observed in the slices of the animal's brain submitted to SE characterizing reactive/hypertrophic astrocytes with large cell bodies and thick cytoskeletal processes, specifically in the basolateral and baso-medial nuclei (Fig. 3). The optical densitometry analysis showed intense GFAP staining for the group E when compared with the group C (C: 0.101 ± 0.002; E: 0.123 ± 0.002; t = 9.193; df = 10; p < 0.0001, unp-TT). The regions with intense reactive astrocyte staining were not limited to the amygdaloid nuclei. GFAP staining around the damaged tissue with a high incidence of lipofuscin was observed in several thalamic nuclei, hippocampus, and paralimbic cortex (data not shown).

## Discussion

Taking into account that the changes induced by the SE involve particularly the ones associated with the inflammatory state,^3^,^16^,^17^ the authors investigated mechanisms that are intrinsically associated with the ionic homeostasis of the brain: NKCC1, KCC2 and Na^+^/K^+^-ATPase. Changes in these mechanisms represent changes that may be directly related to the neuronal excitability state. The present results suggest that the changes observed in the expression of KCC2 and Na^+^-K^+^/ATPase mean an intense change in the chloride regulation of the amygdaloid complex of the animals submitted to SE with pilocarpine. Moreover, lesions in the amygdaloid complex were also observed, mainly in the basolateral and baso-medial nuclei with astrogliosis and cellular debris deposition like lipofuscin. All of these changes are known to promote seizures and also to interfere with the normal functioning of the regions involved.

Severe insults of brain tissue (traumatic lesion, ischemic lesion, or epileptic seizures) may lead to exacerbated glutamate release and intracellular calcium increase with consequent activation of signaling cascade of the cellular death, unleashed by excitotoxicity.^18^ After SE, the reorganization of the neuronal circuitry and of the glial substrate of the injured region may lead to an unbalancing of the inhibitory/excitatory neurotransmission and contributes to the neuronal synchronism and therefore for the seizure threshold decrease,^19^ which justifies what the authors observed.

**Fig. 4:**
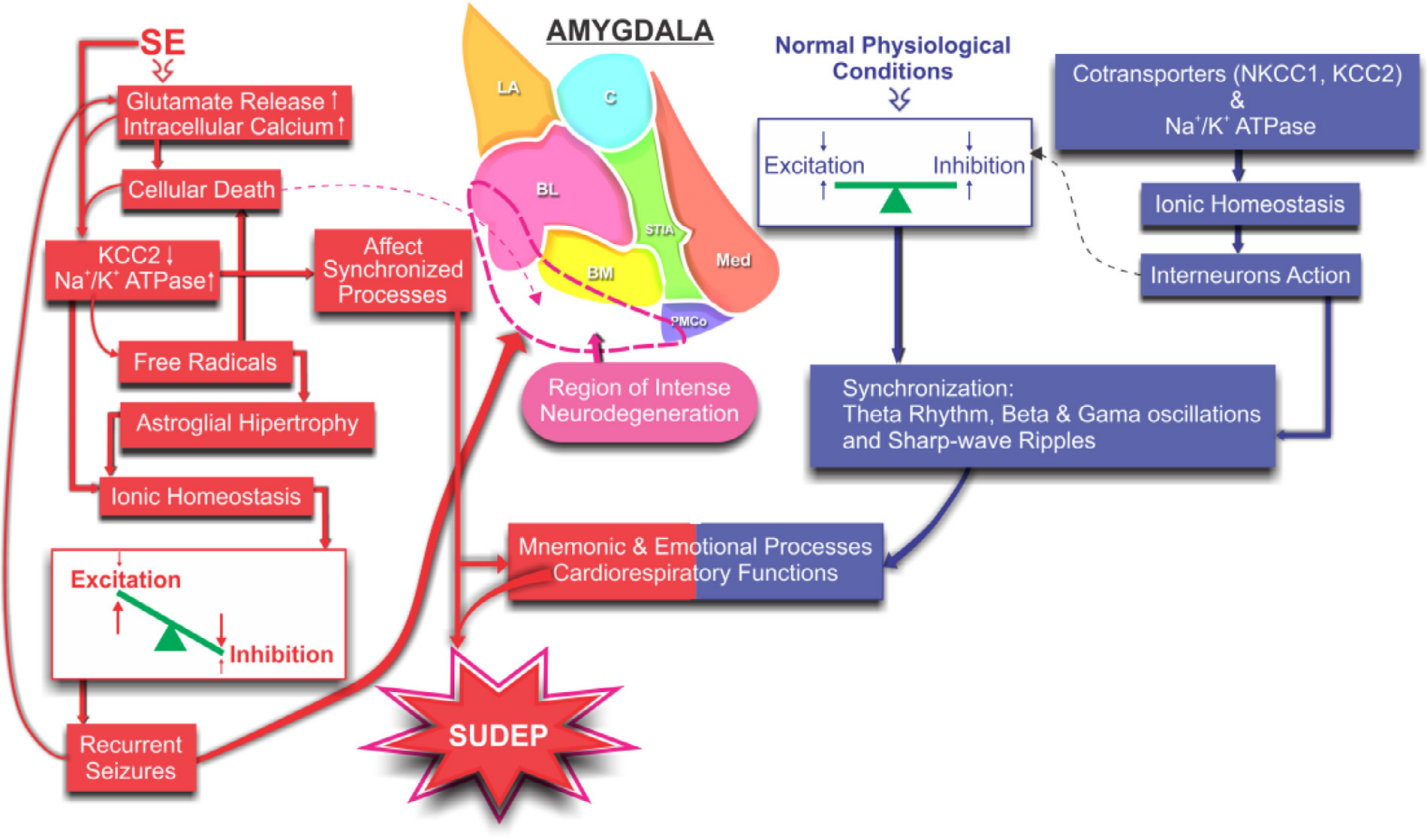
Schematic illustration of the proposed mechanisms involved in the non-synaptic modulation of the ionic homeostasis and the consequent excitation/inhibition balancing during synchronizing processes involving the amygdaloid nucleus: normal (blue) and lesioned by the SE (red). Changes in ionic homeostasis and the consequent unbalancing between excitation and inhibition promote intense neuronal death leading to a proper scenario for spontaneous seizures. More seizures, more intense become progressive degeneration compromising the synchronization processes responsible for cognitive and cardiorespiratory functions, increasing susceptibility to SUDEP.

The large area of tissue damage observed in the piriform cortex, as evidenced in Fig. 4, and with a strong glial reactivity, is related to the predisposition of this region to the action of chemical proconvulsants, such as GABAergic antagonists and muscarinic agonists, such as pilocarpine. The piriform cortex has been classified as the “Tempestas Area”, a critical epileptogenic trigger zone, with a low threshold for kindling and propagation of hypersynchronous activity to the hippocampus and amygdala.^20^,^21^

The amygdala, as well as other limbic structures, has been related to mnemonic and emotional processes.^22^ Even in normal physiological conditions, such processes demand synchronization (theta rhythm, beta and gamma oscillations, and sharp-wave ripples) of some limbic circuits.^23^ This means that the excitation and inhibition must be rigorously regulated by means of the interneurons' action, which, in turn, is highly dependent on the intra and extracellular ionic regulation, preventing excessive synchronisms (hyper-synchronisms) as typically in the SE.

The fast-spiking interneurons have been shown to be responsible for the synchronization processes of pyramidal cell populations in the cortex,^24^,^25^ hippocampus,^26^ and amygdala.^27^ Due to their high interconnectivity through chemical synapses^28^ and gap junctions,^29^ these cells can generate, from afferent excitation, Inhibitory Postsynaptic Potentials (IPSP's) synchronized in thousands of pyramidal cells, generating the so-called tonic inhibition.^24^, ^25^, ^26^ However, it is known that GABA_A_ receptor-mediated GABA_A_ potentials (E_GABAA_) may become depolarizing because of variations in non-synaptic mechanisms responsible for chloride homeostases, such as KCC2 and NKCC1 cotransporters.^30^,^31^

Fast-spiking interneurons in the basolateral nucleus of the amygdala present different GABAergic responses to pyramidal cells,^27^ considered as regular-spiking, raising the hypothesis that these cell types present different mechanisms of intracellular chloride control. From the pharmacological inhibition of NKCC1, with the diuretic bumetanide, a hyperpolarized E_GABAA_ was observed in fast-spiking interneurons, but with no effect on regular-spiking cells. In contrast, KCC2 blockade with furosemide depolarizes E_GABAA_ in regular-spiking neurons, without significant changes in fast-spiking interneurons. This verification allows inferring that, during periods of synchronized network activity, when neurons are depolarized to levels near the threshold, GABAergic neurotransmission should favor the synaptic recruitment of fast-spiking cells in each rhythmic cycle, which would not occur with the regular-spiking cells, controlling the synchronization system under normal conditions. However, with the loss of KCC2, as this investigation has shown throughout the amygdaloid structure of the animals submitted to SE, the amygdala synchronizing processes can go out of order. The regular-spiking neurons, which are more numerous and more dependent on KCC2-mediated control of the internal chloride concentration, would be closer to the firing threshold caused by depolarizing E_GABAA_ and, in turn, would more easily take part in synchronous firing cycles, or would break the local timing of the injured region.

According to Kaila and collaborators,^5^ in the injured region, where the seizures are triggered, the pre-ictal activity leads to a loss of hyperpolarization through the IPSP's, resulting (in the short term) from the high influx of Cl^−^,^32^ which is reinforced and largely consolidated by post-translational downregulation of membrane-bound KCC2^33^,^34^ and subsequently by the blockage of the KCC2 transcription.^33^ In the amygdala, the decrease in KCC2, observed in the animals of the E group 60 days after the SE, may be accompanied by hyperpolarizing IPSPs, leading in turn to local desynchronization, forming an environment conducive to recurrent spontaneous crises such as pointed out by Jiruska et al. (2013).^35^

The strong immunoreactivity to GFAP, which the authors observed in a wide extension of the amygdaloid nuclei of the animals of group E, evidencing the presence of reactive astrogliosis, can also help to explain the reduction of the cotransporter KCC2. Especially after intense inflammatory processes, it has been observed that reactive glial cells promote BDNF secretion.^36^,^37^ BDNF, in turn, activates the Tropomyosin-related kinase B receptor (TrkB), which was directly associated with the downregulation of KCC2 and the blockade of its transcription.^33^,^38^

The up-regulation of NKCC1 expression is another factor that could contribute to the intracellular accumulation of chloride and, therefore, to the synchronization process. However, the authors did not observe up-regulation of NKCC1 in the amygdala nuclei. In the hippocampus, up-regulation of NKCC1 was detected after SE induced by pilocarpine,^39^ which was also observed in this preparation, restricted to the hippocampal region of the animals of group E (data not shown).

Reactive astrogliosis may also be another factor responsible for KCC2 cotransporter changes in group E. Astrocytic reactivity can induce an accumulation of extracellular glutamate, due to the inability to reuptake that amino acid by these altered cells.^37^ According to Kahle et al. (2013),^40^ glutamate plays an important role in the KCC2 dephosphorylation processes and downregulation of the expression of this cotransporter on the membrane.

Reduction of membrane KCC2 may be directly related to the increase in Na^+^/K^+^-ATPase expression observed in group E. Some studies have shown that Na^+^/K^+^-ATPase and KCC2 are functionally linked, acting together to form the structural ion-transport metabolon.^5^ With the decrease of KCC2 expression, and an increase in neuronal activity, an increase in the expression of Na^+^/K^+^-ATPase may occur, due to a compensatory effect. In fact, it has also been observed in the hippocampus that increased activity of this enzyme is in the chronic phase of the pilocarpine model.^41^

The increase in the action of Na^+^/K^+^-ATPase can lead directly to greater production of free radicals, from the oxidative processes of the breakdown of ATP.^42^,^43^ The lipofuscin accumulation that the authors observed in the slices of the animals of the epileptic group sustains the hypothesis of intense oxidative stress. Reactive Oxygen/Nitrogen Species (ROS/RNS) produced during inflammatory processes lead to mitochondrial dysfunction and mitochondrial DNA damage.^44^ This, in turn, affects the synthesis of several enzymatic complexes that are involved in the electron transport chain. The resultant effect includes a cascade of neuroinflammatory reactions, such as lipid peroxidation, astroglial hypertrophy, limbic structure neurodegeneration, neural network reorganization, and hypersynchronism.^44^ These factors predispose the brain to generate recurrent and spontaneous seizures, generally refractory to treatment, being able to propagate from the amygdaloid nuclei through their connections to other encephalic structures like the hypothalamus, pons and medulla.^44^,^45^ These structures are directly related to the control of cardio-respiratory functions and of several autonomous nuclei. In fact, the recruitment of other regions from crises originating from the amygdala or by stimulation of the lateral and basolateral nuclei results in transient apnea.^1^,^46^,^47^ From this observation it becomes evident that these conditions predispose the brain to intractable recurrent seizures and, therefore, to an increased risk of SUDEP. This hypothesis is strengthened by the work of Thom et al.^48^ who demonstrated a high astrocytic density in the amygdaloid nuclei of individuals affected by SUDEP in comparison to non-epileptic controls.

## Conclusion

The findings presented revealed that SE-induced lesion promoted changes in the expression of KCC2 and α1-Na+/K+-ATPase meaning intense change in the chloride regulation in the amygdaloid complex. These changes may contribute to cardiorespiratory dysfunction and may contribute to SUDEP.

### Authors' contributions

Luiz E.C. Santos: Experiments; data analysis; writing the original draft.

Antônio-Carlos G. Almeida: Conceptualization, supervision, writing – review & editing.

Sílvia C.B. Silva: Experiments and data analysis.

Antônio M. Rodrigues: Formal analysis, writing – review & editing.

Samyra G. Cecílio: Experiments and data analysis.

Carla A. Scorza: Review & editing.

Josef Finsterer: Writing – review & editing.

Marcelo Moret: Review & editing.

Fulvio A. Scorza: Conceptualization, data curation, formal analysis.

## Declaration of Competing Interest

The authors declare no conflicts of interest.
